# Growth differentiation factor 6 and transforming growth factor-beta differentially mediate mesenchymal stem cell differentiation, composition, and micromechanical properties of nucleus pulposus constructs

**DOI:** 10.1186/ar4505

**Published:** 2014-03-12

**Authors:** Louise E Clarke, James C McConnell, Michael J Sherratt, Brian Derby, Stephen M Richardson, Judith A Hoyland

**Affiliations:** 1 Centre for Tissue Injury and Repair, Institute of Inflammation and Repair, Faculty of Medical and Human Sciences, The University of Manchester, Oxford Road, Manchester M13 9PT, UK; 2 Wellcome Trust Centre for Cell-Matrix Research, Faculty of Life Sciences, The University of Manchester, Manchester, UK; 3 School of Materials, Faculty of Engineering and Physical Sciences, The University of Manchester, Manchester, UK; 4 NIHR Manchester Musculoskeletal Biomedical Research Unit, Central Manchester University Hospitals NHS Foundation Trust, Manchester Academic Health Science Centre, Nowgen Building, 29 Grafton Street, Manchester M13 9WU, UK

## Abstract

**Introduction:**

Currently, there is huge research focus on the development of novel cell-based regeneration and tissue-engineering therapies for the treatment of intervertebral disc degeneration and the associated back pain. Both bone marrow-derived (BM) mesenchymal stem cells (MSCs) and adipose-derived MSCs (AD-MSCs) are proposed as suitable cells for such therapies. However, currently no consensus exists as to the optimum growth factor needed to drive differentiation to a nucleus pulposus (NP)-like phenotype. The aim of this study was to investigate the effect of growth differentiation factor-6 (GDF6), compared with other transforming growth factor (TGF) superfamily members, on discogenic differentiation of MSCs, the matrix composition, and micromechanics of engineered NP tissue constructs.

**Methods:**

Patient-matched human AD-MSCs and BM-MSCs were seeded into type I collagen hydrogels and cultured in differentiating media supplemented with TGF-β3, GDF5, or GDF6. After 14 days, quantitative polymerase chain reaction analysis of chondrogenic and novel NP marker genes and sulfated glycosaminoglycan (sGAG) content of the construct and media components were measured. Additionally, construct micromechanics were analyzed by using scanning acoustic microscopy (SAM).

**Results:**

GDF6 stimulation of BM-MSCs and AD-MSCs resulted in a significant increase in expression of novel NP marker genes, a higher aggrecan-to-type II collagen gene expression ratio, and higher sGAG production compared with TGF-β or GDF5 stimulation. These effects were greater in AD-MSCs than in BM-MSCs. Furthermore, the acoustic-wave speed measured by using SAM, and therefore tissue stiffness, was lowest in GDF6-stiumlated AD-MSC constructs.

**Conclusions:**

The data suggest that GDF6 stimulation of AD-MSCs induces differentiation to an NP-like phenotype and results in a more proteoglycan-rich matrix. Micromechanical analysis shows that the GDF6-treated AD-MSCs have a less-stiff matrix composition, suggesting that the growth factor is inducing a matrix that is more akin to the native NP-like tissue. Thus, this cell and growth-factor combination may be the ideal choice for cell-based intervertebral disc (IVD)-regeneration therapies.

## Introduction

Low back pain (LBP), is an increasing socioeconomic burden in today’s society. Current therapies involve conservative symptomatic pain relief or end-stage surgical treatments. However, these therapies are relatively unsuccessful in the long term and do not address the underlying pathogenesis of LBP, such as IVD degeneration, which correlates with LBP in 40% of cases [1].

Degenerative changes occur predominantly in the highly hydrated central nucleus pulposus (NP) which is composed of the proteoglycan, aggrecan, and type II collagen. With degeneration, degradation of the extracellular matrix (ECM) occurs, with substantial loss of aggrecan [2]. These changes result in dehydration of the ECM, thereby influencing tissue stiffness and strength, which leads to a reduction in the structural integrity of the disc, ultimately compromising its function [3]. Given the poor long-term efficacy of current clinical interventions, research is now focused on cell-based tissue-engineering strategies. Such strategies aim to target the underlying pathogenesis by replacing the cell population and thereby restoring a functional IVD matrix. Of these approaches, minimally invasive implantation of mesenchymal stem cell (MSC)-seeded hydrogels offers the most promise.

Both bone marrow- and adipose-derived MSCs (BM-MSCs and AD-MSCs, respectively) are able to differentiate into NP-like cells [4-6]. Native adult NP cells are conventionally described as being ”chondrocyte-like” and characterized through their rounded morphology and expression of classic chondrogenic markers, including *SOX-9*, type II collagen, and aggrecan [7]. However, the composition of the NP and articular cartilage ECM is significantly different, with NP tissue having a substantially more-proteoglycan-rich ECM than has cartilage [8]. Thus, if MSCs are to be used for IVD regeneration, it is essential to identify accurately the differentiated cell phenotype and to ensure appropriate ECM synthesis.

Our discovery of novel human NP cell markers (*CAXII*, *FOXF1*, *KRT 8*, *18*, *19*) [9] has allowed a clear distinction to be made between the chondrocyte and NP cell phenotype. We previously demonstrated that these markers can be used to identify accurately both BM-MSC and AD-MSC differentiation toward NP-like cells [6]. This work used culture in a three-dimensional (3D) environment with media containing TGF-β, a growth factor more commonly used to induce chondrogenesis, and focused on gene and protein marker expression by differentiated cells. However, although such studies demonstrate that MSCs can be induced to adopt an NP-like phenotype and hence synthesize an ECM matrix that, in biochemical terms, is compositionally similar to native NP tissue, they provide no information on the structural and biomechanical properties of the synthesized matrix. Importantly, the mechanical behavior of ECM-rich tissues is determined not only by the identity of the molecular components but also by their posttranslational modification and assembly into structurally malleable macromolecular aggregates [10-12]. Therefore, although TGF-β has been commonly used by several groups to induce NP-like differentiation, the effects of alternative growth factors should be characterized with regard to cell phenotype, protein synthesis, and crucially local stiffness to optimize the biochemical and biomechanical characteristics of the resultant construct.

Growth differentiation factor 5 (GDF5/BMP-14/CDMP-1) and growth differentiation factor 6 (GDF6/BMP-13/CDMP-2) are members of the TGF superfamily and are associated with skeletal development [13-15]. We previously demonstrated that they are expressed by human NP cells and that GDF5 increases type II collagen and aggrecan gene expression by degenerate human NP cells *in vitro*[16]. GDF5 has also recently been shown to improve differentiation of BM-MSCs to an NP-like phenotype (that is, discogenic differentiation) compared with TGF-β1, as shown by the enhanced expression of the novel markers *CAXII*, *KRT19*, and *FOXF1*[17-19]. However, whereas differentiation to NP-like cells was achieved, GDF5 did not produce a more-proteoglycan (PG)-rich ECM than did TGF-β1. As such, and given that GDF5 has also been shown to induce chondrogenic differentiation [20], this may not be the optimal growth factor for directing MSC differentiation to an NP-like cell capable of synthesizing an ECM with appropriate biochemical and biomechanical characteristics.

GDF6 has been shown to play an important role in spinal column development, implying that it may have a pivotal role in IVD development and homeostasis [21]. Furthermore, research has shown that injection of GDF6 prevents IVD degeneration in an experimentally induced ovine model [22]. Taken together, this suggests that GDF6 may be an appropriate growth factor to drive MSC differentiation toward an NP-like cell and hence to produce a PG-rich ECM more akin to native IVD tissue than that produced by TGF-β- or GDF5-stimulated MSCs.

Thus, we hypothesized that the choice of exogenous growth factor and MSC source would influence cell differentiation, ECM composition, and mechanical stiffness of engineered NP tissue constructs. Differentiation of both BM-MSCs and AD-MSCs after stimulation with TGF-β, GDF5, and GDF6 was assessed by expression of classic and novel NP marker genes and PG production. Additionally, by using scanning acoustic microscopy, the effect of exogenous growth factors on micromechanical stiffness of tissue constructs was assessed, with acoustic wave speed serving as a surrogate measure of tissue stiffness [23,24].

## Methods

### Mesenchymal stem cell culture

Human patient-matched AD-MSCs and BM-MSCs were isolated from subcutaneous fat and bone marrow removed from the proximal femur during hip-replacement surgery after approval from the North West Research Ethics Committee and fully informed written consent of patients (*n* = 7; average age, 47 years (age range, 22 to 78 years); four women, three men). BM-MSCs were isolated and expanded in α-MEM with 20% fetal calf serum (hereafter termed standard media), as previously described [25]. After 5 days, nonadherent cells were discarded, and adherent cells were cultured to confluence.

To isolate AD-MSCs, adipose tissue was digested in collagenase solution (30 mg collagenase type I in Hanks Balanced Salt Solution (HBSS) and 20 m*M* calcium chloride) for 2 hours at 37°C. The solution was filtered, neutralized with standard media, and centrifuged for 5 minutes. Supernatant was aspirated, and cells cultured to confluence in standard media, with nonadherent cells discarded after 5 days.

The CD profile of BM-MSCs and AD-MSCs was analyzed by using flow cytometry and multipotentiality assessed along the three mesenchymal lineages by using standard methods (data not shown). Cells at passage 3 were used for subsequent experiments.

### Encapsulation of MSCs in type I collagen hydrogels

Collagen gels were established by combining 3 mg/ml atelosoluble type I collagen (Devro, Edinburgh, Scotland) (pH 2), neutralization buffer (0.2 *M* sodium phosphate, 1.3 *M* sodium chloride, pH 11.2) and αMEM at an 8:1:1 ratio, respectively. MSCs were suspended in the collagen solution at room temperature to a final cell density of 4 × 10^6^/ml and 100 μl gels formed in 0.4-μm high-density cell-culture inserts (BD Biosciences, San Jose, CA, USA). Gels were cultured for 24 hours in standard media, and media were subsequently replaced with a differentiating medium, as defined later, either with or without growth factor.

### MSC pellet cultures

MSCs were dispensed into a 15-ml Falcon tube at a density of 250,000 cells in 2 ml of standard media. Subsequently cells were centrifuged, incubated at 37°C for 24 hours, and media replaced with a differentiating media either with or without the respective growth factor.

### Differentiation of MSCs with TGF-β3, GDF5, and GDF6

MSCs were encapsulated in type I collagen gels and cultured in differentiating media consisting of high-glucose DMEM, 1% FCS, insulin-transferrin-selenium (ITS-X) (Gibco, Grand Island, NY, USA), 100 μ*M* ascorbic acid-2-phosphate, 1.25 mg/ml bovine serum albumin (BSA), 10^−7^ *M* dexamethasone, 5.4 μg/ml linoleic acid, 40 μg/ml L-proline, and 100 U/ml penicillin, 100 μg/ml streptomycin, and 2.5 μg/ml amphotericin B. To assess optimal growth-factor concentration, media were further supplemented with either no growth factor (control), TGF-β3 (Invitrogen) at concentrations of 1, 10, 100 ng/ml, GDF5 (PeproTech, Rocky Hill, NJ, United States) at concentrations of 10, 100, or 1,000 ng/ml, or GDF6 (PeproTech) at concentrations of 10, 100, or 1,000 ng/ml for 14 days. Concentration ranges were chosen to encompass manufacturers’ recommendations and previously published concentrations [18,26,27].

After this assessment, cells were encapsulated in type I collagen gels or pellets and cultured in differentiating media supplemented with either no growth factor or optimal concentrations of growth factor for 14 days. Media were changed every 48 hours and retained for subsequent analysis of sulfated glycosaminoglycan (sGAG) content.

### Assessment of NP marker gene expression with quantitative real-time PCR

After 14 days, cell-seeded collagen hydrogels and pellets were disrupted with Molecular Grinding Resin in TRIzol (Geno Technology Inc., St. Louis, MO, USA), and RNA was extracted according to the manufacturer’s recommendations. cDNA was generated by using a high-capacity cDNA reverse-transcription kit (Life Technologies) and diluted to 5 ng/μl. Gene expression was analyzed with quantitative real-time PCR by using an Applied Biosystems StepOne Plus Real Time PCR system. Reactions were prepared in triplicate by using LuminoCt qPCR readymix reagents (Sigma-Aldrich, Irvine, UK) to a total volume of 10 μl, containing 10 ng cDNA, 900 n*M* each primer, and 250 n*M* probe. Data were analyzed according to the 2^-ΔΔ^ Ct method, with expression normalized to the average of two prevalidated housekeeping genes (*EIF2B1* and *MRPL19*) and to the control sample [28].

### Assessment of PG sGAG content

sGAG production was assessed in triplicate by using a dimethylmethylene blue (DMMB) assay, as previously described [29,30], and quantified against a chondroitin sulfate C (shark cartilage, Sigma) standard curve. Media collected at each media change were also used to analyze the PG content released into media on a cumulative basis.

Total DNA content was measured by using a Quant-iT PicoGreen assay (Life Technologies) with a lambda DNA standard curve, according to manufacturer’s instructions. sGAG values in gels/pellets were normalized to dsDNA content.

### Histologic characterization of ECM deposition

Collagen constructs were embedded in OCT; snap-frozen in liquid nitrogen, and cryosectioned to a nominal thickness of 5 μm. Safranin-O/Fast Green staining was performed to localize sGAG according to standard protocols. Picrosirius red staining and polarized light microscopy were used to assess fibrillar collagen content, as previously described [31]. Fibrillar collagen content was assessed by imaging the same area under bright-field and cross-polarized light microscopy. Total tissue area was compared with birefringent fibrillar collagen area, and percentage fibrillar collagen content calculated (these calculations were based on overall intensity of staining and did not distinguish between color of staining, which subjectively shows fibril diameter) [31]. Three areas were assessed per section, from each of three sections per cell type and growth-factor treatment.

### Micromechanical characterization by scanning acoustic microscopy

The SAM imaging technique has been described in detail previously [11,24,32]. Imaging of hydrated cryosections was performed by using a KSI 2000 microscope (PVA TePla Analytical Systems GmbH; Herborn, Germany) with bespoke data acquisition and control systems. SAM data were collected in triplicate by using the Multi-Layer Phase Analysis (MLPA) method [24] with a scan size of 200 μm and a frequency of 770 MHz to produce an acoustic wave speed map.

### Statistical analysis

The nonparametric Mann–Whitney *U* test was applied to determine significance to compare gene expression, sGAG, and DNA. Values are reported as mean ± SEM. Analysis of SAM data was conducted by using SPSS 20 (SPSS, Chicago, IL, USA). Q-q plots were used to test for normality, and one-way ANOVAs were used to compare means. For all analyses, a value of *P* < 0.05 was considered statistically significant.

## Results

### Optimal growth factor concentration

**Figure 1:**
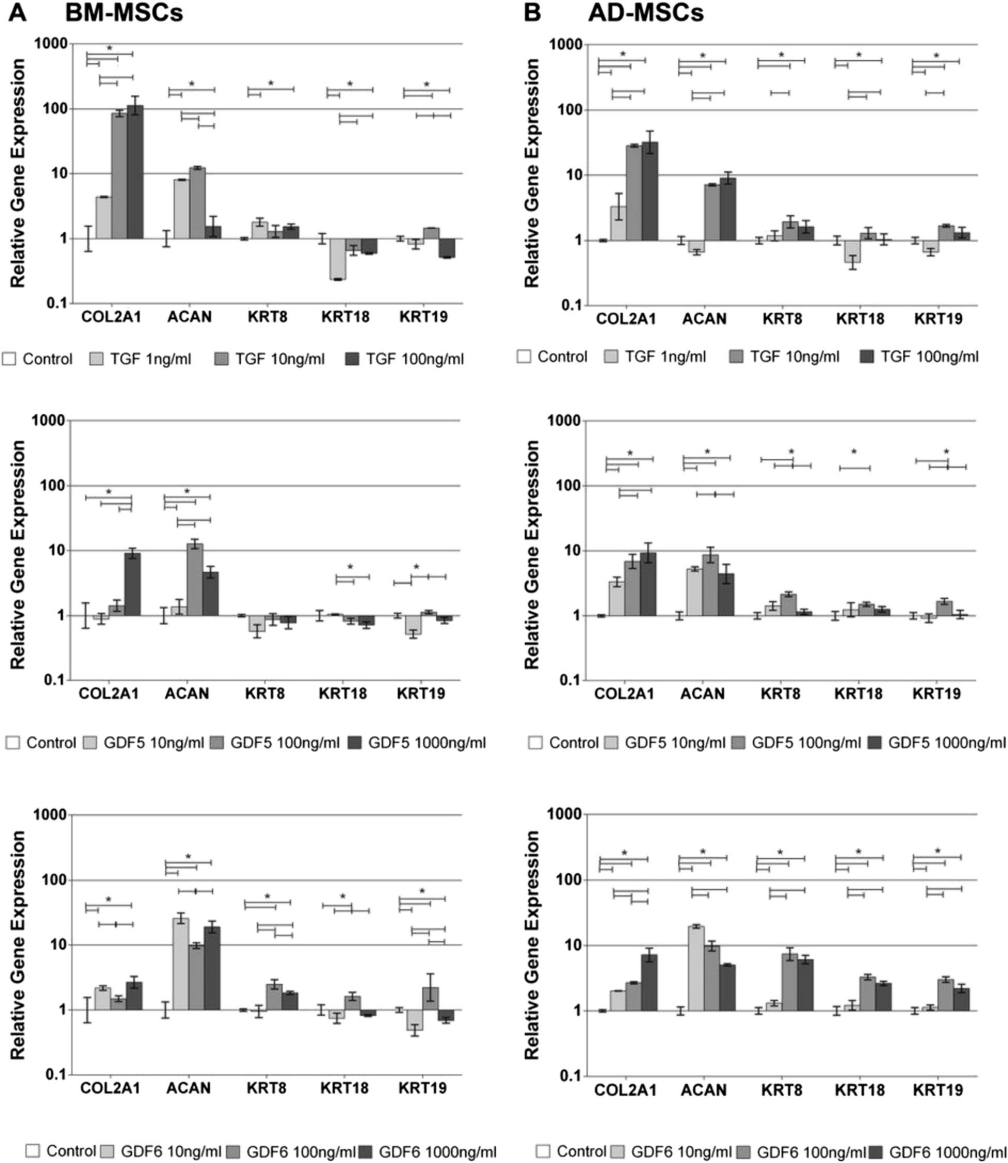
**Quantitative real-time PCR analysis of gene expression in response to varying concentrations of growth factors. (A)** BM-MSCs and **(B)** AD-MSCs were seeded in type I collagen gels and stimulated with varying concentrations of TGF-β, GDF5, or GDF6 for 14 days. Relative gene expression was normalized to mean housekeeping-gene expression and cells without growth-factor stimulation and plotted on a log scale. *N* = 3; data represent mean ± SEM. *P < 0.05.

When compared with no growth factor controls, both BM-MSCs (Figure 1A) and AD-MSCs (Figure 1B) demonstrated dose-dependent responses to all growth factors. Both *COL2A1* and *ACAN* gene expressions were significantly upregulated with all growth factors, whereas novel maker gene expression was significantly upregulated after GDF6 stimulation, in particular in AD-MSCs. Overall, the optimal expression profiles were identified at 10 ng/ml TGF-β and 100 ng/ml of GDF5 and GDF6.

### Conventional NP marker expression in type I collagen hydrogels

**Figure 2:**
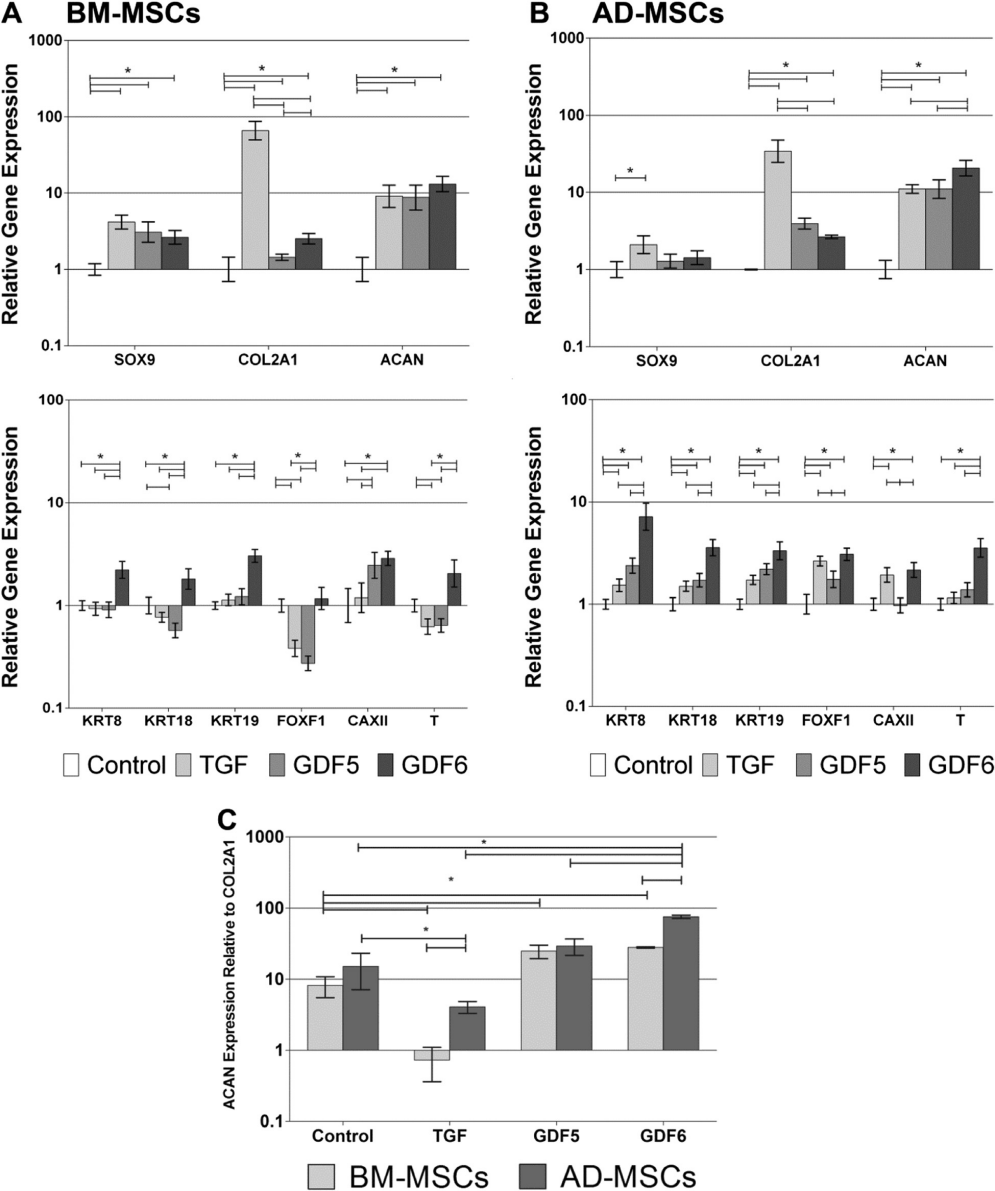
**Quantitative real-time PCR analysis of gene-expression changes in response to growth factor stimulation. (A)** BM-MSCs, and **(B)** AD-MSCs were cultured for 14 days in type I collagen hydrogels and stimulated with optimal concentrations of TGF-β, GDF5, or GDF6. Relative gene expression was normalized to mean housekeeping-gene expression and cells without growth factor stimulation and plotted on a log scale. **(C)** Aggrecan-to-type II collagen gene expression ratio in BM and AD-MSCs after culture in type I collagen hydrogels for 14 days with either no growth factor, or optimal concentrations of TGF-β, GDF5, or GDF6. Relative gene expression was normalized to mean housekeeping-gene expression and plotted on a log scale. *N* = 7; all data represent mean ± SEM. *P < 0.05.

Culture of BM-MSCs in the presence of all growth factors (Figure 2A) resulted in a significant upregulation of *SOX9* and *ACAN* compared with the control, with the largest upregulation of *ACAN* (13-fold) being identified in the GDF6-stimulated cohort. Conversely, culture with TGF-β demonstrated the greatest increase in expression of *COL2A1* (66-fold compared with control) compared with treatment with either GDF5 (1.5-fold) or GDF6 (2.5-fold). Culture of AD-MSCs in the presence of all growth factors (Figure 2B) resulted in a significant increase in *ACAN* expression compared with control, with GDF6 again causing a significantly greater increase (20-fold) than other growth-factor treatments. As with BM-MSCs, TGF-β treatment of AD-MSCs resulted in the largest upregulation of *COL2A1* (34-fold) gene expression.

When the *ACAN*-to-*COL2A1* relative gene expression was assessed (2^-ΔCtACAN-ΔCtCOL2A1^), AD-MSCs stimulated with GDF6 demonstrated the highest ratio of 75:1, whereas BM-MSCs demonstrated a ratio of 29:1 (Figure 2C). Cells treated with TGF-β demonstrated the lowest ratio overall at 4:1 for AD-MSCs and 0.7:1 for BM-MSCs.

### Novel NP marker expression in type I collagen hydrogels

Treatment of BM-MSCs with TGF-β and GDF5 caused no change in *KRT8*, *KRT19*, or *CAXII* and a down regulation of *KRT18*, *FOXF1*, and *T* gene expression compared with controls (Figure 2A). Conversely, treatment with GDF6 significantly upregulated *KRT8*, *18*, *19*, *CAXII*, and *T*, with no change noted in *FOXF1* compared with controls. Culture of AD-MSCs with GDF6 resulted in significant upregulation of all novel marker genes compared with the control, and in the case of *KRT8*, *KRT18*, *KRT19*, and *T* expression, was significantly higher than that that seen in AD-MSCs cultured with either TGF-β or GDF5 (Figure 2B). Comparison between cell types showed that expression levels were consistently upregulated to a greater extent in AD-MSCs than in BM-MSCs.

### SGAG content

**Figure 3:**
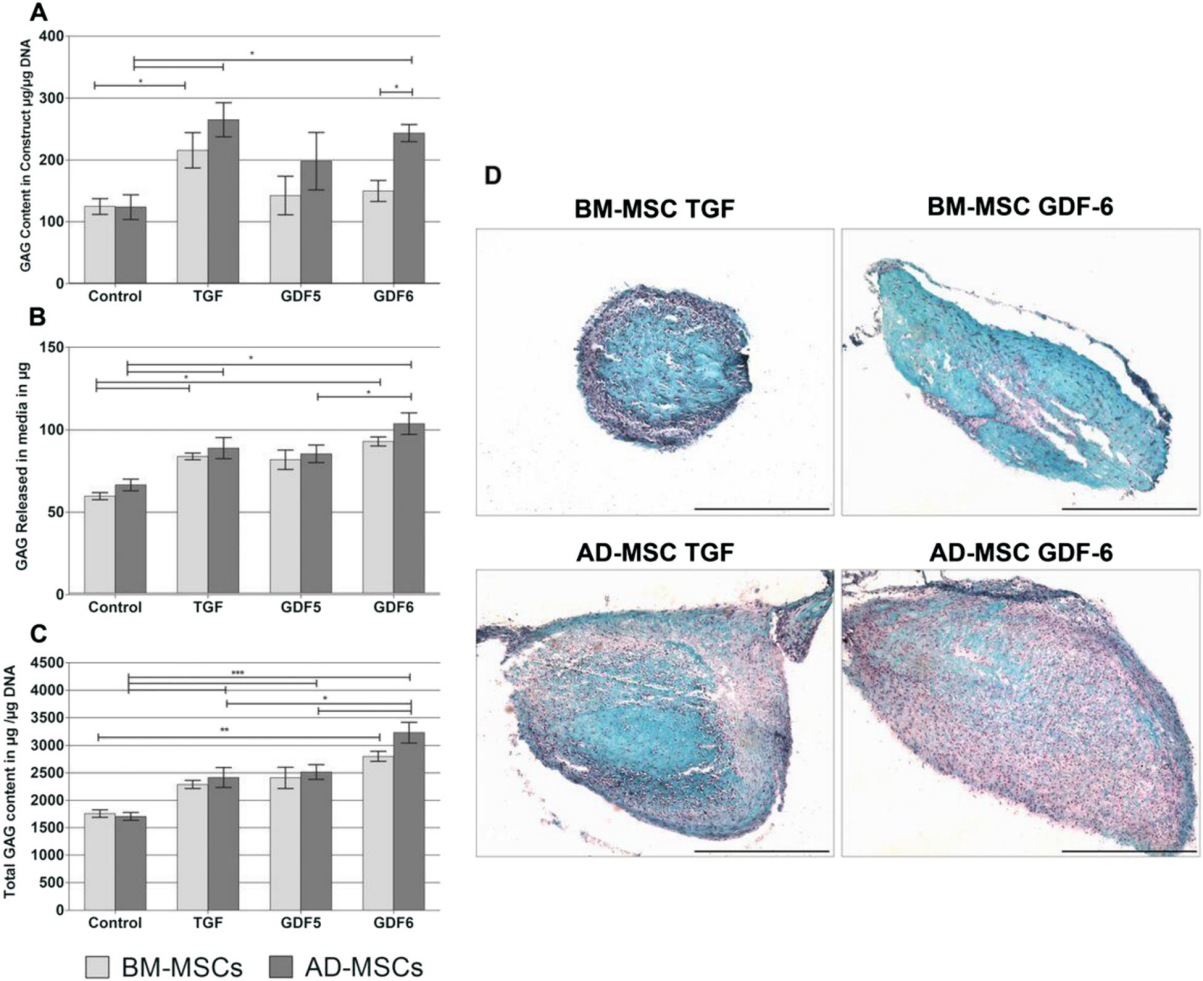
**Analysis of sGAG production by BM and AD-MSCs in response to growth-factor stimulation.** BM and AD-MSCs were seeded in type I collagen hydrogels and cultured in the absence or presence of TGF-β, GDF5, or GDF6. **(A)** DMMB quantification of sGAG retained within gel constructs, normalized to DNA content within the construct. **(B)** DMMB quantification of sGAG released cumulatively into the media throughout the culture period. **(C)** Quantification of total sGAG content in the construct and media normalized to DNA content within the construct at day 14. *N* = 3, data represent mean ± SEM. *P < 0.05. **(D)** Safranin O staining of MSC-seeded collagen gels demonstrating deposition of sGAG throughout the constructs, with AD-MSCs stimulated with GDF6 having the highest and most homogeneous sGAG deposition. Scale bars, 500 μm.

AD-MSCs in type I collagen hydrogels showed significant increases in the sGAG/DNA content within the constructs after stimulation with TGF-β (265.00 μg ±27.71) and GDF6 (243.53 μg ± 13.87), whereas BM-MSCs displayed significant increases only after TGF-β stimulation (215.59 μg ± 28.74) compared with controls (Figure 3A). AD-MSCs consistently demonstrated higher levels of sGAG/DNA compared with BM-MSCs, significantly so in the case of GDF6 stimulation (149.60 μg ±17.05 in BM-MSCs versus 243.53 μg ± 13.87 in AD-MSCs).

The sGAG released into the media over 14 days (Figure 3B) was highest in AD-MSCs treated with GDF6 (103.69 μg ± 8.5 μg), although BM-MSCs and AD-MSCS treated with either TGF-β or GDF6 consistently demonstrated significant increases in released sGAG compared with the control.

The combined total sGAG/DNA from both construct and media showed that AD-MSCs treated with GDF6 produced the most sGAG (3,228.62 μg ± 185.98), and this was significantly higher than the control group (1,703.74 μg ± 72.89), or after treatment with either TGF-β (2,414.88 μg ± 180.79) or GDF5 (2,512.97 μg ± 134.44). Likewise, BM-MSCs synthesized the most sGAG after GDF-6 treatment (2,799.28 μg ±92.33), although this was lower than that produced by AD-MSCs.

Safranin-O staining of type I collagen hydrogels (Figure 3D) demonstrated a more-widespread distribution of sGAG within the GDF6-stimulated AD-MSC constructs compared with TGF-β-stimulated constructs. BM-MSCs treated with TGF-β showed staining confined to the periphery of the construct only, whereas AD-MSCs treated with either TGF-β or GDF6 demonstrated a more-homogeneous distribution throughout the construct.

### Fibrillar collagen content and local stiffness

**Figure 4:**
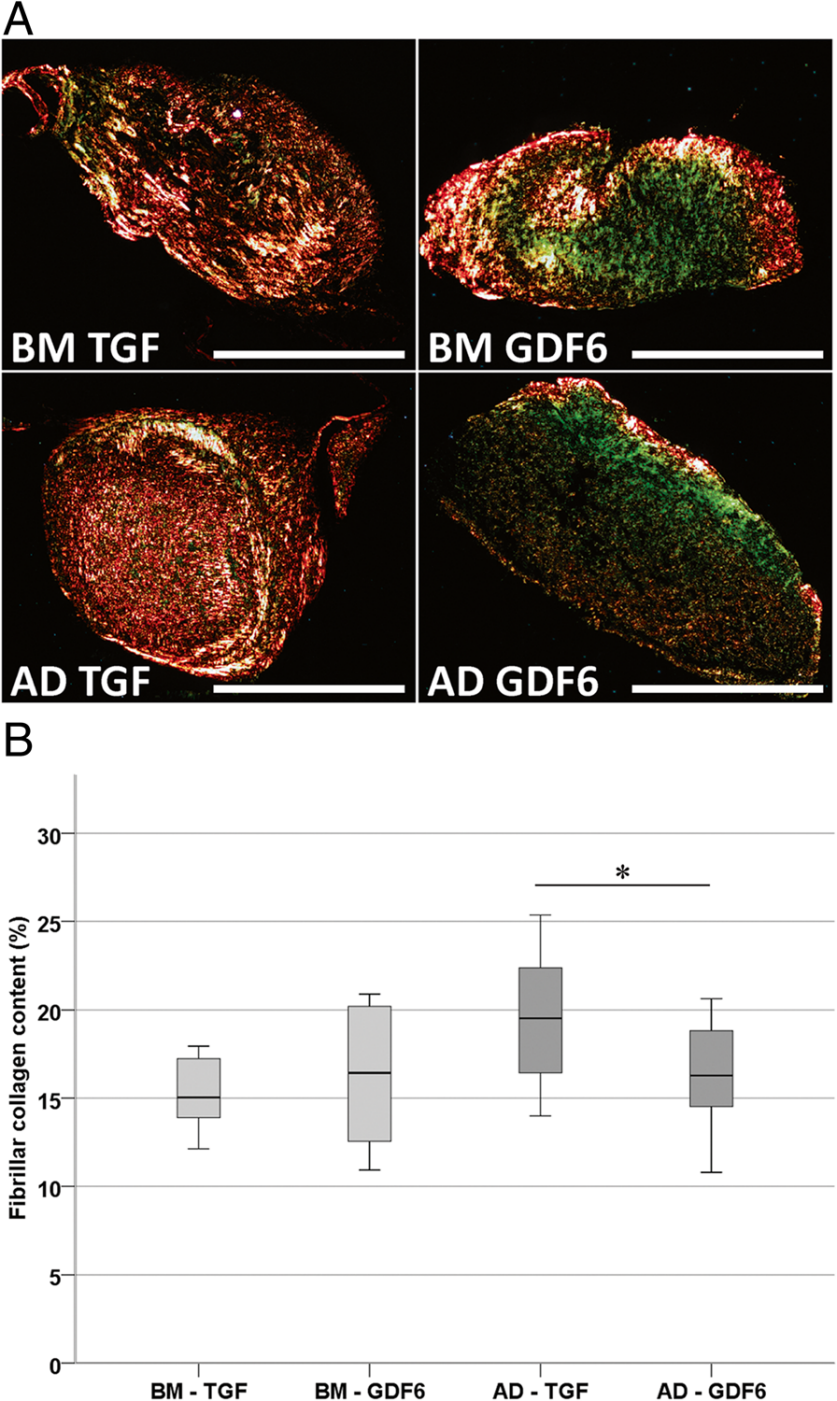
**Fibrillar collagen content visualized by using polarized light microscopy of picrosirius red-stained sections. (A)** Picrosirius red staining of MSC-seeded type I collagen hydrogels stimulated with TGF-β or GDF6, demonstrating enhanced fibrillar collagen deposition (red and green stains) in AD-MSC-seeded constructs stimulated with TGF-β compared with GDF6-stimulated constructs. Scale bar, 500 μm. **(B)** Quantification of percentage fibrillar collagen content in BM-MSC- and AD-MSC-seeded constructs after stimulation with TGF-β or GDF6. AD-MSC-seeded constructs stimulated with TGF-β demonstrated a significantly higher percentage of fibrillar collagen content compared with GDF-6-stimulated constructs; *N* = 3.

**Figure 5:**
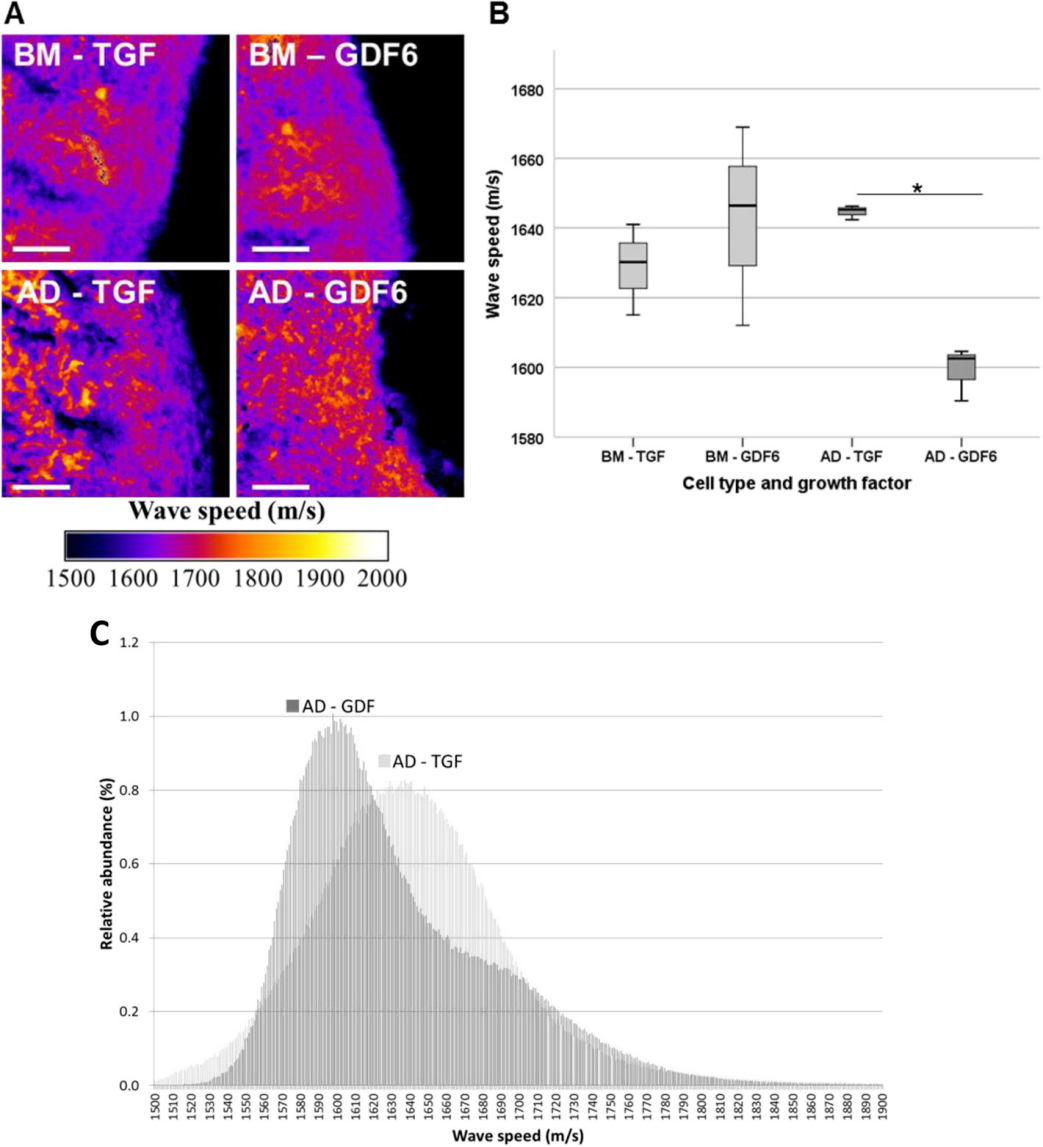
**Mean acoustic wave speed (a surrogate measure of tissue stiffness) assessed with scanning acoustic microscopy. (A)** Acoustic wave-speed distribution maps of BM-MSC- and AD-MSC-seeded type I collagen hydrogels stimulated with TGF-β or GDF6. Scale bar, 50 μm. **(B)** Quantification of wave speed in BM-MSC- and AD-MSC-seeded constructs after stimulation with TGF-β or GDF6. GDF6 stimulation of AD-MSCs resulted in a significantly decreased acoustic-wave speed compared with TGF-β-stimulated constructs, suggesting that GDF6-stimulated AD-MSC produced a less-stiff ECM. *N* = 3. *P < 0.05. **(C)** Wave-speed distribution in AD-MSC-seeded constructs after stimulation with either TGF-β or GDF6. The GDF6-stimulated construct shows significantly reduced mean wave speed of 1,599 ms^−1^ compared with the TGF-β-stimulated counterpart, 1,644 ms^−1^.

Fibrillar collagen deposition by BM-MSCs was independent of growth factor species (Figure 4; TGF-β, 15.4% ± 0.6%; GDF6, 16.0% ± 1.07%). In contrast, exposure to exogenous TGF-β and GDF6 induced significant differences in the deposition of fibrillar collagens by AD-MSCs (Figure 4; TGF-β, 19.5% ± 0.9%; GDF6, 16.2% ± 0.7%). These MSC source and growth factor-related differences in fibrillar collagen content were, in turn, correlated with the mean acoustic wave speed (and hence stiffness) of the tissue constructs. Whereas mean acoustic wave speed in TGF-β- and GDF6-treated BM-MSC constructs was growth factor independent (Figure 5; TGF-β, 1,629 ms^−1^ ± 8 ms^−1^; GDF6, 1,642 ms^−1^ ± 17 ms^−1^), acoustic-wave speed was significantly higher in TGF-β-treated AD-MSC seeded constructs compared with their GDF6-treated counterparts (Figure 5; TGF-β, 1,644 ms^−1^ ± 1 ms^−1^; GDF6, 1,599 ms^−1^ ± 4 ms^−1^).

### MSC differentiation in pellet culture

**Figure 6:**
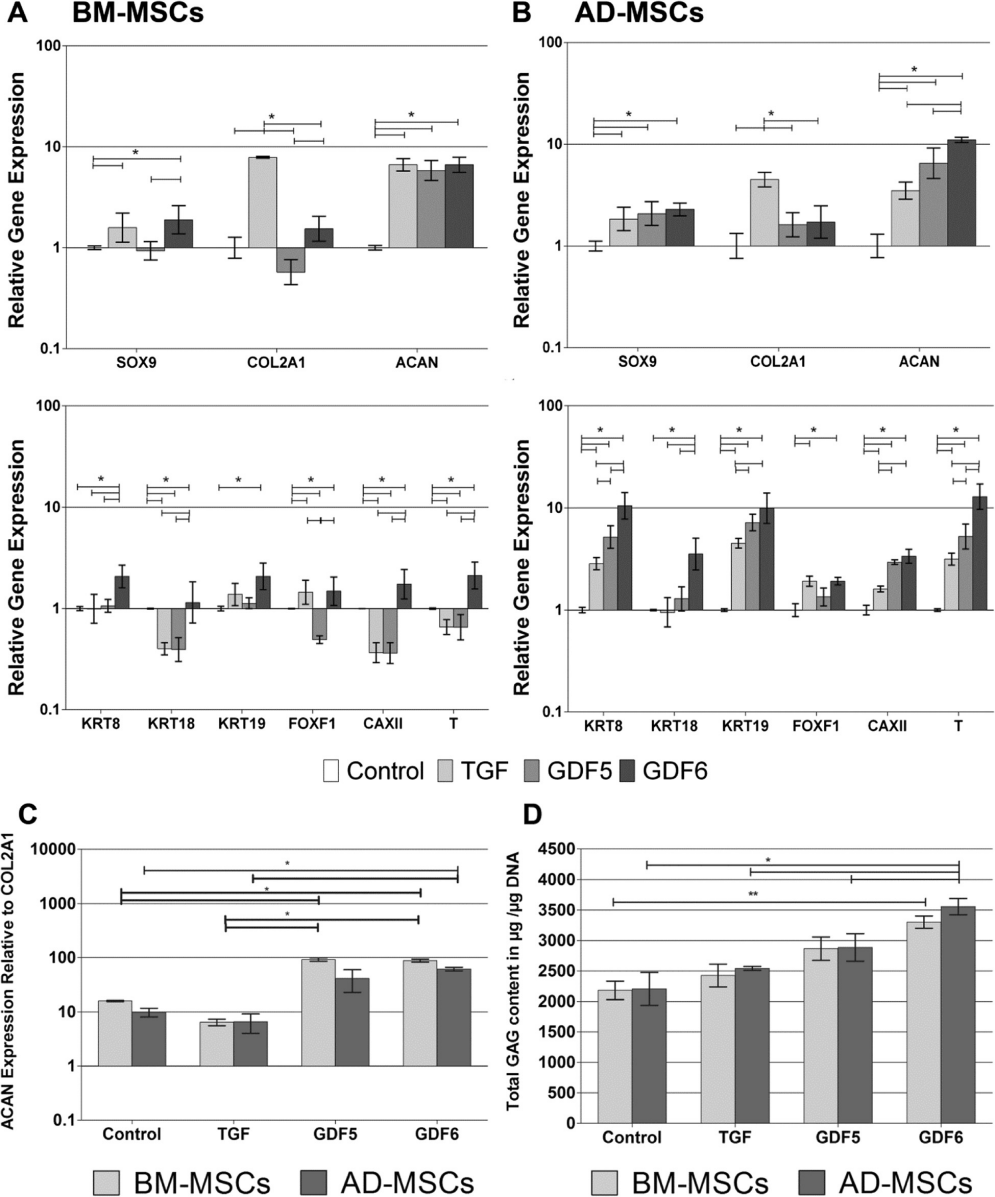
**Analysis of BM and AD-MSC response to growth-factor stimulation in pellet culture.** Quantitative real-time PCR analysis of gene-expression changes in **(A)**, BM-MSCs and **(B)**, AD-MSCs in pellets in the absence or presence of TGF-β, GDF5, or GDF6. Relative gene expression was normalized to mean housekeeping-gene expression and cells without growth-factor stimulation and plotted on a log scale. **(C)** Aggrecan-to-type II collagen gene-expression ratio in BM and AD-MSCs after culture in type I collagen hydrogels for 14 days with either no growth factor, TGF-β, GDF5, or GDF6. Relative gene expression was normalized to mean housekeeping-gene expression and plotted on a log scale. **(D)** DMMB quantification of sGAG content within pellets and cumulative release into media over 14 days normalized to DNA content of the pellet at day 14. For all analyses, *N* = 3; data represent mean ± SEM. *P < 0.05.

To control for any influence of the reconstituted type I collagen hydrogels on differentiation, MSCs were cultured in three-dimensional pellets. Levels of conventional NP marker-gene expression (Figure 6A, B) were similar to that seen previously, with AD-MSCs showing the highest upregulation of *ACAN* after GDF6 stimulation, whereas TGF-β induced the largest increase in *COL2A1* expression in both cell types. BM-MSCs demonstrated either no change or a downregulation of novel NP marker genes after TGF-β or GDF5 stimulation, with GDF6 causing significant upregulation of *KRTs 8*, *18*, and *19*, *FOXF1*, *CAXII*, and *T* (as with type I collagen hydrogels). In AD-MSCs, GDF6 caused a significant upregulation of all marker genes, with increases in *KRT8*, *18*, *19*, *CAXII*, and *T* being significantly higher after GDF6 stimulation than with either TGF-β or GDF5.

The ratio of *ACAN*-to-*COL2A1* in BM-MSCs and AD-MSCs was higher in both GDF5 and GDF6 groups compared with either controls or TGF-β-treated cells, with no significant difference identified between the two cell types after stimulation with any of the growth factors (Figure 6C).

Total GAG/DNA from both pellets and accumulated media showed that AD-MSCs treated with GDF6 had the highest total value (3,556.31 μg ± 133.06), and this was significantly higher than both the control (2,206.34 μg ± 267.54), and after treatment with either TGF-β (2,540.32 μg ± 32.78) or GDF5 (2,886.65 μg ± 225.76) (Figure 6D).

## Discussion

Given the similarities in gene-expression profiles between articular chondrocytes and NP cells, specifically expression of *SOX-9*, type II collagen, and aggrecan, researchers have increasingly investigated the potential of MSCs to regenerate the IVD. However, to date, studies have applied methods more commonly used to induce chondrogenesis, specifically, culture in a 3D environment and differentiating media containing TGF-β and thereafter using ECM gene and protein expression as outcome measures to depict an NP-like tissue.

However, the differences in matrix composition between articular cartilage and NP tissue, together with recent phenotypic profiling studies comparing their native cells, suggest that these outcome measures are insufficient to define the end-stage differentiated cell and that more-detailed analyses are needed. Given the increased understanding of the NP cell phenotype, unique phenotypic markers can now be used to define accurately the lineage-specific MSC differentiation toward NP-like cells. This has allowed alternative culture systems, in particular, comparison of different growth factors, to be investigated with the aim of optimizing MSC discogenic differentiation and ensuring synthesis of an ECM with appropriate biochemical and biomechanical properties.

Members of the TGF-β superfamily are potential candidates for driving discogenic differentiation, with a recent study showing that GDF5 induces differentiation of MSCs *in vitro* to a more-IVD-like phenotype than TGF-β [17-19]. Given that we previously demonstrated expression of GDF5 and GDF6 by human NP cells [16] and that both AD-MSCs and BM-MSCs can differentiate to NP-like cells [6], here we aimed to investigate whether GDF6 may be a more-appropriate stimulus for MSC discogenic differentiation and production of an NP-like ECM than either TGF-β or GDF5.

Stimulation of both AD-MSCs and BM-MSCs with GDF6 resulted in increased expression of novel NP phenotypic marker genes *KRT8*, *18*, and *19*, *FOXF1* and *CAXII*, compared with either TGF-β or GDF5, particularly in AD-MSCs. Given that this panel of markers has been identified as NP specific and previously used to depict appropriate differentiation [6,9] , the observed increases in gene expression suggest that both AD-MSCs are BM-MSCs are capable of discogenic differentiation, which is particularly enhanced by GDF6.

We also investigated the expression of the notochordal and mesodermal marker brachyury (*T*), which has previously been shown to be expressed by adult NP cells [9] and demonstrated the greatest induction of gene expression after GDF6 stimulation, again particularly in AD-MSCs. Importantly, these findings were consistent with both cell types in both type I collagen hydrogel and pellet cultures, suggesting that the biomaterial did not influence differentiation, with growth-factor choice having the predominant effect on discogenic differentiation.

The molecules aggrecan and type II collagen are key components of the IVD ECM, with a higher aggrecan-to-type II collagen ratio (both at the gene transcription [17] and protein [8] level being indicative of an NP-like, rather than articular cartilage-like phenotype/matrix). As expected [32,33], stimulation of both BM-MSCs and AD-MSCs with TGF-β resulted in the highest *COL2A1* gene expression and lowest *ACAN*-to-*COL2A1* ratio. When combined with the decrease or small upregulation of novel NP marker genes in BM-MSCs and AD-MSCS, respectively, and the increased expression of *SOX-9* (major transcription factor for chondrogenesis), the data suggest that TGF-β is driving differentiation more toward a chondrocyte-like phenotype. Conversely, as was previously reported, GDF5 promoted *ACAN* gene expression, and resulted in an increased *ACAN*-to-*COL2A1* ratio compared with TGF-β stimulation [17,18]. Although stimulation of BM-MSCs with GDF6 resulted in a similar response to that of GDF5, AD-MSCs stimulated with GDF6 produced significantly higher levels of *ACAN* and demonstrated the highest *ACAN*-to-*COL2A1* ratio, indicative of an NP-like phenotype. Importantly, this gene-expression analysis (as assessed by using both conventional and novel NP markers, as well as *ACAN*-to-*COL2A1* ratio) has shown that GDF6 promotes discogenic differentiation of both BM-MSCs and AD-MSCs, with gene-expression changes being greatest in AD-MSCs, suggesting that these cells may be more able to differentiate to an NP-like phenotype.

Further to characterize these differences between the differentiated cell populations and to ensure that changes in gene expression were reflected at the protein level, analysis of sGAG synthesis was assessed. Cells under all conditions synthesized detectable levels of sGAG, with significant increases in retained sGAG (normalized to DNA) seen after TGF-β stimulation in both BM-MSCs and AD-MSCs, and after both TGF-β and GDF6 stimulation in AD-MSCs. A substantial amount of sGAG was also released into the media by all cells, with significant increases again being seen in both cell types after stimulation with either TGF-β or GDF6. Whereas the majority of newly synthesized sGAG was not retained within the constructs, similar findings have been observed in other comparable studies [19,34]. This may be due to the fiber density of the matrix produced, with differences in collagen synthesis or remodeling of the collagen hydrogel altering matrix retention.

However, when total sGAG synthesis was analyzed and normalized to DNA content, significantly more sGAG was synthesized by AD-MSCs after stimulation with all three growth factors, with AD-MSCs stimulated with GDF6 producing the most sGAG. Interestingly, GDF5 did not result in significant increases in sGAG synthesis in either cell type, a finding that has been reported for BM-MSCs previously [18]. As with the gene-expression profile, DMMB analysis revealed similar results after pellet culture, with GDF6-stimulated BM- and AD-MSCs synthesizing the largest amount of sGAGs. Interestingly, safranin-O staining of the collagen constructs also highlighted differences, whereas AD-MSCs stimulated with either TGF-β or GDF6 demonstrated more-intense safranin O staining than BM-MSCs, supporting the DMMB analysis on retained sGAG.

Additionally, a homogeneous GAG distribution was observed throughout the construct compared with the more-focal/discrete staining in BM-MSC constructs. Overall, given that GDF6-stimulated AD-MSCs produce the highest levels of sGAG and the most homogeneous matrix deposition, this cell type and stimulation may be the more-appropriate choice for regeneration of the PG-rich NP, whereas TGF-β may promote a more-articular cartilage-like matrix.

Although the majority of studies to date have relied on molecular and biochemical methods to characterize the ability of tissue-construct systems to mimic the target tissues, the micro- and hence macromechanical behavior of the construct is likely to influence the efficacy of any resulting therapy. Therefore, in this study, we quantified the acoustic-wave speed [23] of tissue-construct cryosections (which is proportional to the square root of the material’s Young modulus (stiffness)) [35]. Having previously used SAM to localize age-related changes in vascular stiffness [24], here we demonstrate that a refined version of this technique (MLPA) is able to distinguish cell- and cytokine-specific effects on the resultant micromechanical behavior of tissue constructs. Furthermore, these effects are in concordance with our other findings that AD-MSC-seeded constructs treated with GDF6 showed decreased fibrillar collagen and increased proteoglycan deposition when compared with their TGF-β-treated counterparts.

AD-MSCs are thought to be better candidates for cell therapy because of their ease of access, limited donor-site morbidity, and high proliferation rate. Importantly, this study demonstrated differences in regenerative potential for IVD application between BM-MSCs and AD-MSCs, with the data suggesting that AD-MSCs are more able to undergo discogenic differentiation and produce an appropriate PG-like matrix. It was previously reported that AD-MSCs have a reduced capacity to differentiate to a chondrogenic lineage compared with BM-MSCs [36] because of differences in receptor expression (specifically, TGF-β receptor I [36]) compared with BM-MSCS. As such, the differential effects of the exogenous growth factors used here (with TGF-β stimulating more of a chondrogenic phenotype and GDF-6, an NP- like phenotype and matrix) may be due to differences in cell-signaling pathways despite all being members of the TGF-β superfamily. TGF-β3 is recognized by type II (TGFRII) and type I surface receptors (ALK5, ALK1), which in turn facilitate the Smad 2/3 signaling pathway.

Conversely, GDF5 and GDF6 use BMPRII, ActRIIa, ALK3, and ALK6 receptors activating the alternative Smad 1/5/8 pathway [37]. Activation of these distinct signaling pathways may then lead to different downstream signals, which may explain the more-chondrogenic nature of TGF-β3 stimulation. GDF5 and GDF6 are 82% homologous in the highly conserved active C-terminal (mature signaling) domain [38] and are thus likely to operate by similar ligand/receptor interactions. However, recent evidence reporting differential effects of these factors on mouse calvarial osteogenesis [39] would suggest that these growth factors may have distinct signaling effects, despite similar receptor use, which could also account for the differential effects seen here, although elucidation of the signaling mechanisms is beyond the scope of this article.

## Conclusion

The phenotype of differentiated cells, together with the ECM composition and micromechanics of tissue-engineered constructs, must be extensively characterized to ascertain that an appropriately functioning tissue is formed. In this study, we demonstrated differentiation of MSCs to an NP-like phenotype and formation of appropriate NP ECM components. Interestingly, AD-MSCs treated with GDF6 produced a less-stiff, PG-rich matrix, which may be more indicative of the native healthy NP ECM.

## Abbreviations

AD-MSC: Adipose-derived mesenchymal stem cell; BM-MSC: bone marrow-derived mesenchymal stem cell; BMP: bone morphogenic protein; DMMB: dimethylmethylene blue; ECM: extracellular matrix; GDF5: growth differentiation factor 5; GDF6: growth differentiation factor 6; IVD: intervertebral disc; LBP: low back pain; NP: nucleus pulposus; PG: proteoglycan; SAM: scanning acoustic microscopy; sGAG: sulfated glycosaminoglycan; TGF: transforming growth factor.

## Competing interests

All authors state that no conflict of interests exists.

## Authors’ contributions

LEC, Conception and design, collection and/or assembly of data, data analysis and interpretation, manuscript writing. JCM, conception and design, collection and/or assembly of data, data analysis and interpretation, manuscript writing. MJS, conception and design, data analysis and interpretation, manuscript writing. BD, data analysis and interpretation, critical revision, final approval of manuscript. SMR, co-investigator for securing funding, conception and design, data analysis and interpretation, manuscript writing, final approval of manuscript. JAH, secured funding, conception, and design, data analysis and interpretation, manuscript writing, final approval of manuscript. All authors read and approved the final manuscript.
